# Statistical analysis plan for the Balanced Solution *versus* Saline in Intensive Care Study (BaSICS)

**DOI:** 10.5935/0103-507X.20200081

**Published:** 2020

**Authors:** Lucas Petri Damiani, Alexandre Biasi Cavalcanti, Rodrigo Santos Biondi, Flávio Geraldo Rezende de Freitas, Rodrigo Cruvinel Figueiredo, Wilson José Lovato, Cristina Prata Amêndola, Ary Serpa Neto, Jorge Luiz da Rocha Paranhos, Viviane Cordeiro Veiga, Marco Antonio Vieira Guedes, Eraldo de Azevedo Lúcio, Lúcio Couto Oliveira Júnior, Thiago Costa Lisboa, Fabio Holanda Lacerda, Tamiris Abait Miranda, Israel Silva Maia, Cintia Magalhães Carvalho Grion, Flavia Ribeiro Machado, Fernando Godinho Zampieri

**Affiliations:** 1 Research Institute, HCor-Hospital do Coração - São Paulo (SP), Brazil.; 2 Instituto de Cardiologia do Distrito Federal, Fundação Universitária de Cardiologia - Brasília (DF), Brazil.; 3 Hospital e Maternidade SEPACO - São Paulo (SP), Brazil.; 4 Hospital e Maternidade São José - Colatina (ES), Brazil.; 5 Hospital das Clínicas, Faculdade de Medicina de Ribeirão Preto, Universidade de São Paulo - Ribeirão Preto (SP), Brazil.; 6 Hospital de Amor - Barretos (SP), Brazil.; 7 Hospital Israelita Albert Einstein - São Paulo (SP), Brazil.; 8 Santa Casa de Misericórdia de São João Del-Rei - São João Del-Rei (MG), Brazil.; 9 BP - A Beneficência Portuguesa de São Paulo - São Paulo (SP), Brazil.; 10 Hospital Ana Nery – Salvador (BA), Brazil.; 11 Hospital São Francisco, Santa Casa de Misericórdia de Porto Alegre - Porto Alegre (RS), Brazil.; 12 Hospital Geral Cleriston Andrade - Feira de Santana (BA), Brazil.; 13 Hospital Santa Rita, Santa Casa de Misericórdia de Porto Alegre - Porto Alegre (RS), Brazil.; 14 Hospital da Luz - São Paulo (SP), Brazil.; 15 Hospital Nereu Ramos - Florianópolis (SC), Brazil.; 16 Hospital Universitário Regional do Norte do Paraná - Londrina (PR), Brazil.; 17 Hospital São Paulo, Escola Paulista de Medicina, Universidade Federal de São Paulo - São Paulo (SP), Brazil.

**Keywords:** Balanced solutions, Critical care, Normal saline, Saline solution, Acute kidney injury, Soluções balanceadas, Terapia intensiva, Solução salina normal, Solução salina, Lesão renal aguda

## Abstract

**Objective:**

To report the statistical analysis plan (first version) for the Balanced Solutions *versus* Saline in Intensive Care Study (BaSICS).

**Methods:**

BaSICS is a multicenter factorial randomized controlled trial that will assess the effects of Plasma-Lyte 148 *versus* 0.9% saline as the fluid of choice in critically ill patients, as well as the effects of a slow (333mL/h) *versus* rapid (999mL/h) infusion speed during fluid challenges, on important patient outcomes. The fluid type will be blinded for investigators, patients and the analyses. No blinding will be possible for the infusion speed for the investigators, but all analyses will be kept blinded during the analysis procedure.

**Results:**

BaSICS will have 90-day mortality as its primary endpoint, which will be tested using mixed-effects Cox proportional hazard models, considering sites as a random variable (frailty models) adjusted for age, organ dysfunction and admission type. Important secondary endpoints include renal replacement therapy up to 90 days, acute renal failure, organ dysfunction at days 3 and 7, and mechanical ventilation-free days within 28 days.

**Conclusion:**

This manuscript provides details on the first version of the statistical analysis plan for the BaSICS trial and will guide the study’s analysis when follow-up is finished.

## Abstract (pt)

**Objetivo:**

Relatar o plano de análise estatística (primeira versão) para o estudo *Balanced Solutions versus Saline in Intensive Care Study* (BaSICS).

**Métodos:**

O estudo BaSICS é um ensaio multicêntrico fatorial e randomizado que avaliará os efeitos da administração dos fluidos Plasma-Lyte 148 em comparação com solução salina 0,9% como fluido de escolha em pacientes críticos, assim como os efeitos de uma velocidade de infusão lenta (333mL/hora) em comparação com uma velocidade de infusão rápida (999mL/hora) durante desafios com volume, em importantes desfechos do paciente. O tipo de fluido será mantido cego para os investigadores, pacientes e nas análises. Não será possível, entretanto, ocultar dos investigadores a velocidade de infusão, mas os procedimentos de análise serão mantidos cegos quanto a esse aspecto.

**Resultados:**

O estudo BaSICS terá como parâmetro primário a mortalidade em 90 dias, que será testada com utilização de modelos de risco proporcional de Cox de efeitos mistos, considerando os centros de estudo como variável randômica (modelos de fragilidade) ajustada por idade, disfunção de órgãos e tipo de admissão. Os parâmetros secundários importantes incluem terapia de substituição renal até 90 dias, insuficiência renal aguda, disfunção de órgãos nos dias 3 e 7 e dias sem ventilação mecânica em 28 dias.

**Conclusão:**

Este artigo fornece detalhes referentes à primeira versão do plano de análise estatística para o estudo BaSICS e orientará a análise do estudo após a conclusão do seguimento.

## INTRODUCTION

The Balanced Solutions *versus* Saline in Intensive Care Study (BaSICS) Trial is a multicenter, randomized, factorial, clinical trial that will assess the effects of Plasma-Lyte 148 *versus* 0.9% saline as the fluid of choice in critically ill patients, as well as the effects of a slow (333mL/h) *versus* rapid (999mL/h) infusion speed during fluid challenges, on important patient outcomes. The original protocol paper emphasizing the importance of the study, and the rationale and inclusion/exclusion criteria has been published previously, but details of the statistical approach and reporting of obtained data were not described.^(1)^

This report aims to provide details of the BaSICS statistical analysis plan (SAP), based on protocol version 3. There are several challenges in designing a statistical analysis plan for this study due to its factorial design, the potential exclusion of patients after enrollment expected with the use of posterior consent, and the interplay between survival and intervention use. Finally, it is expected that some important mediators are involved in the possible effects of both interventions, and this SAP represents an opportunity to define, a priori, how they will be analyzed and reported. The main study´s hypothesis, for both arms, is that a balanced solution and slow infusion speed can reduce 90-day mortality in critically ill patients. This statistical analysis plan for the BaSICS trial aims to prevent statistical analysis bias arising from exploratory analyses after the study results are known. It was prepared before the end of follow-up for all included patients by an independent statistical analyst without knowledge of the interim analysis conducted by the data monitoring committee.

### Study background and flowchart

**Figure 1:**
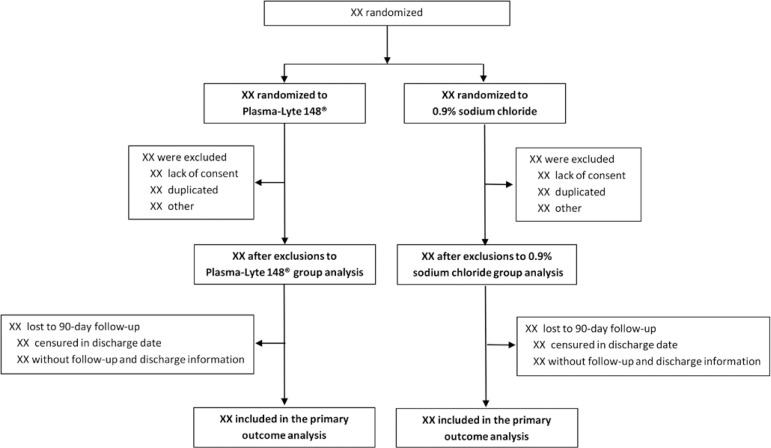
Plasma-Lyte *versus* saline.

**Figure 2:**
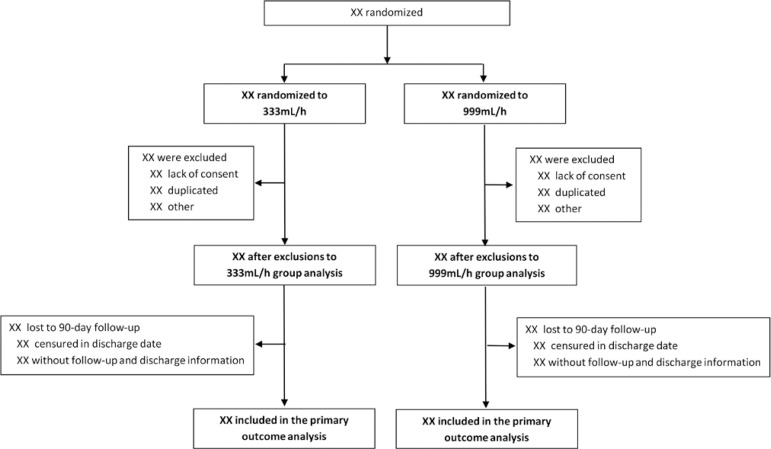
Infusion speed.

BaSICS is a large pragmatic critical trial that aims to provide evidence for or against the use of Plasma-Lyte 148 over 0.9% saline and of lower infusion speeds during fluid challenges in critically ill patients. Patients were randomized using a web-based system designed for the trial in a factorial way (blocks of 12 patients), stratified by recruiting site in a 1:1:1:1 ratio to both define a specific fluid group (Plasma-Lyte 148 or 0.9% saline) and slow (333mL/h) or rapid (999mL/h) infusion during a fluid challenge.^(1)^ The details of the inclusion and exclusion criteria have been addressed in the original protocol, together with recommendations for fluid management and safety rules.^(1)^ The planned flowcharts for both interventions are shown in figures 1 and 2, respectively. BaSICS is a large pragmatic trial, and a screening log was not obtained at each site due to the large number of included patients and for local logistics reasons.

Blinding to fluid type was possible due to identical bags manufactured by Baxter LA® labeled “A” to “F” (six types, with half of the letters saline and half Plasma-Lyte 148). Sites were instructed to use the study fluid for the fluid challenge, maintenance and, whenever possible, for the dilution of all drugs (if compatible) that required an infusion volume over 100mL. Use of fluid bolus and fluid management was left to the discretion of the attending physician due to the pragmatic nature of the trial; however, guidelines and good-clinical practices in fluid management were provided to the sites in the study material and were discussed during the investigator´s meetings. Infusion speed could not be blinded.

The answers to these two questions are important because it is presently unclear whether balanced (low chloride) solutions are beneficial for critically ill patients and because no large clinical trial has ever assessed the effects of varying infusion speeds on robust patient-centered outcomes.

### Sample size calculation, interim analyses and database lock

BaSICS was designed to enroll 11,000 patients. The sample size was calculated estimating 35% mortality within 90 days in the control group (saline and rapid infusion). This was based on data from large Brazilian cohorts, as discussed in the study protocol.^(1)^ The defined sample size allowed for an 89% power to detect a hazard ratio of 0.9 for both interventions assuming no interaction was expected among the study interventions.

Four interim analyses were performed in BaSICS. The first analysis of the first 1,000 patients aimed exclusively at checking the safety of the fluid speed comparison. Three other interim analyses followed at 25% (2,750 patients), 50% (5,500 patients) and 75% of the sample size (8,250 patients). We used very restrictive rules for stopping the trial for efficacy and we shall not adjust p-values for sequential tests (Appendix 1). A database lock will be applied after 90-day follow-up of all patients is obtained and all needed actions to obtain follow-up have been deployed. The baseline features of the included patients will be displayed as in table 1.

**Table 1:** Baseline characteristics of the patients

Characteristics	Plasma Lyte		0.9% Sodium chloride	
	Slow infusion (n = xxx)	Rapid infusion (n = xxx)	Slow infusion (n = xxx)	Rapid infusion (n = xxx)
Age (years)	xx.x ± xx.x	xx.x ± xx.x	xx.x ± xx.x	xx.x ± xx.x
Female sex	xx.x (xx.x)	xx.x (xx.x)	xx.x (xx.x)	xx.x (xx.x)
Weight (kg)	xx.x ± xx.x	xx.x ± xx.x	xx.x ± xx.x	xx.x ± xx.x
Height (m)	xx.x ± xx.x	xx.x ± xx.x	xx.x ± xx.x	xx.x ± xx.x
Source of admission to ICU				
Elective surgery	xx.x (xx.x)	xx.x (xx.x)	xx.x (xx.x)	xx.x (xx.x)
Nonelective surgery	xx.x (xx.x)	xx.x (xx.x)	xx.x (xx.x)	xx.x (xx.x)
Emergency Department	xx.x (xx.x)	xx.x (xx.x)	xx.x (xx.x)	xx.x (xx.x)
Ward	xx.x (xx.x)	xx.x (xx.x)	xx.x (xx.x)	xx.x (xx.x)
Another hospital	xx.x (xx.x)	xx.x (xx.x)	xx.x (xx.x)	xx.x (xx.x)
Another ICU	xx.x (xx.x)	xx.x (xx.x)	xx.x (xx.x)	xx.x (xx.x)
APACHE II	xx.x [xx.x - xx.x]	xx.x (xx.x - xx.x]	xx.x (xx.x - xx.x]	xx.x [xx.x - xx.x]
SOFA	xx.x [xx.x - xx.x]	xx.x [xx.x - xx.x]	xx.x [xx.x - xx.x]	xx.x [xx.x - xx.x]
Mechanical ventilation				
Noninvasive mechanical ventilation > 12 hours	xx.x (xx.x)	xx.x (xx.x)	xx.x (xx.x)	xx.x (xx.x)
Invasive mechanical ventilation	xx.x (xx.x)	xx.x (xx.x)	xx.x (xx.x)	xx.x (xx.x)
Serum creatinine (mg/dL)	xx.x ± xx.x	xx.x ± xx.x	xx.x ± xx.x	xx.x ± xx.x
Cirrhosis or acute liver failure	xx.x (xx.x)	xx.x (xx.x)	xx.x (xx.x)	xx.x (xx.x)
Heart failure	xx.x (xx.x)	xx.x (xx.x)	xx.x (xx.x)	xx.x (xx.x)
Time from ICU admission to randomization (days)	xx.x [xx.x - xx.x]	xx.x [xx.x - xx.x]	xx.x [xx.x - xx.x]	xx.x [xx.x - xx.x]
Balanced crystalloid and saline administration in the 24 hours before enrollment				
Balanced solution				
Proportion of patients who received fluid	xx.x (xx.x)	xx.x (xx.x)	xx.x (xx.x)	xx.x (xx.x)
Fluid volume (mL)	xx.x [xx.x - xx.x]	xx.x [xx.x - xx.x]	xx.x [xx.x - xx.x]	xx.x [xx.x - xx.x]
Saline				
Proportion of patients who received fluid	xx.x (xx.x)	xx.x (xx.x)	xx.x (xx.x)	xx.x (xx.x)
Fluid volume (mL)	xx.x [xx.x - xx.x]	xx.x [xx.x - xx.x]	xx.x [xx.x - xx.x]	xx.x [xx.x - xx.x]
Predefined subgroups				
KDIGO criteria for acute kidney injury ≥ 1	xx.x (xx.x)	xx.x (xx.x)	xx.x (xx.x)	xx.x (xx.x)
Sepsis	xx.x (xx.x)	xx.x (xx.x)	xx.x (xx.x)	xx.x (xx.x)
Traumatic brain injury	xx.x (xx.x)	xx.x (xx.x)	xx.x (xx.x)	xx.x (xx.x)
Surgical patients	xx.x (xx.x)	xx.x (xx.x)	xx.x (xx.x)	xx.x (xx.x)
APACHE II ≥ 25	xx.x (xx.x)	xx.x (xx.x)	xx.x (xx.x)	xx.x (xx.x)
Receipt of > 1000mL of saline in the 24 hours prior to randomization	xx.x (xx.x)	xx.x (xx.x)	xx.x (xx.x)	xx.x (xx.x)

ICU - intensive care unit; APACHE - Acute Physiology and Chronic Health Evaluation; SOFA - Sequential Organ Failure Assessment; MAP - mean arterial pressure; KDIGO - Kidney Disease Improving Global Outcomes. Results expressed as mean ± standard deviation, n (%) or median [interquartile range].

### BaSICS inclusion and exclusion criteria

#### Inclusion criteria

To be randomized, patients must meet all of the following inclusion criteria: need for fluid resuscitation/plasma expansion and the clinician considers that Plasma-Lyte 148 or saline are equally appropriate for the patient, with no specific indications or contraindications for any of the fluids or for rapid or slow infusion; patients are not expected to be discharged on the day after their admission; at least one of the following risk factors for acute kidney injury (AKI):

- Age ≥ 65 years.
- Hypotension (mean arterial pressure - MAP < 65mmHg or systolic blood pressure - SBP < 90mmHg) or the use of vasopressors.
- Sepsis, defined as Sepsis 3 criteria.^(2)^
- Use of invasive mechanical ventilation or of continuous noninvasive mechanical ventilation (including a high-flow nasal cannula) > 12 hours.
- Oliguria (< 0.5mL/kg/hour for ≥ 3 hours).
- Serum creatinine ≥ 1.2mg/dL for women or ≥ 1.4mg/dL for men.
- Liver cirrhosis or acute liver failure.

#### Exclusion criteria

Were excluded: patients age < 18 years; acute renal failure treated with renal replacement therapy (RRT) or expected to require RRT within the next 6 hours; severe electrolyte disturbances (serum sodium ≤ 120mmol/L or ≥ 160mmol/L); death considered imminent and inevitable within 24 hours; with suspected or confirmed brain death; under exclusive palliative care and those previously enrolled in the BaSICS study

### Planned statistical analysis for the primary, secondary and tertiary endpoints

All clinical endpoints shall be presented as similar as possible to those shown in table 2. Categorical variables will be presented as the number and percentage. Continuous variables will be presented as the mean and standard deviation for variables such as age and physiological parameters (blood pressure, sodium and potassium levels). We will favor the presentation of ordinal variables, such as Sequential Organ Failure Assessment (SOFA) score, as the median and interquartile range. An alpha of 5% and 95% confidence intervals will be used unless otherwise stated; similarly, all analyses will be based on the intention-to-treat principle unless otherwise stated. Analyses will be performed with the latest R version available at the end of the patients’ follow-up (most probably version 4.0).

**Table 2:** Outcomes comparing slow versus fast infusion speed and Plasma-Lyte versus 0.9% sodium chloride

Characteristics	Plasma-Lyte	0.9% Sodium chloride	Effect measure	p value*	Slow infusion	Rapid infusion	Effect measure	p value†	p value‡
			(95%CI)		(n = xxx)	(n = xxx)	(95%CI)		
Primary outcome									
90-day mortality	xx.x (xx.x)	xx.x (xx.x)	x.xx (x.xx - x.xx)	x.xx	xx.x (xx.x)	xx.x (xx.x)	x.xx (x.xx - x.xx)	x.xx	x.xx
Secondary outcomes									
Acute renal failure with need for renal replacement therapy within 90 days									
Incidence (per 1000 patient-day)	xx.x	xx.x	x.xx (x.xx - x.xx)	x.xx	xx.x	xx.x	x.xx (x.xx - x.xx)	x.xx	x.xx
At day 1	xx.x (xx.x)	xx.x (xx.x)		-	xx.x (xx.x)	xx.x (xx.x)		-	-
At day 2	xx.x (xx.x)	xx.x (xx.x)		-	xx.x (xx.x)	xx.x (xx.x)		-	-
At day 3	xx.x (xx.x)	xx.x (xx.x)		-	xx.x (xx.x)	xx.x (xx.x)		-	-
At day 7	xx.x (xx.x)	xx.x (xx.x)		-	xx.x (xx.x)	xx.x (xx.x)		-	-
In hospital (at least one renal substitution in hospital stay)	xx.x (xx.x)	xx.x (xx.x)	x.xx (x.xx - x.xx)	x.xx	xx.x (xx.x)	xx.x (xx.x)	x.xx (x.xx - x.xx)	x.xx	x.xx
Unknown at 90-day follow up	xx.x (xx.x)	xx.x (xx.x)		-	xx.x (xx.x)	xx.x (xx.x)		-	-
Lost contact for 90-day follow-up	xx.x (xx.x)	xx.x (xx.x)		-	xx.x (xx.x)	xx.x (xx.x)		-	-
Incident renal failure (using KDIGO ≥ 2) at day 3	xx.x (xx.x)	xx.x (xx.x)	x.xx (x.xx - x.xx)	x.xx	xx.x (xx.x)	xx.x (xx.x)	x.xx (x.xx - x.xx)	x.xx	x.xx
KDIGO ≥ 2 or death at day 3	xx.x (xx.x)	xx.x (xx.x)	x.xx (x.xx - x.xx)	x.xx	xx.x (xx.x)	xx.x (xx.x)	x.xx (x.xx - x.xx)	x.xx	x.xx
Incident renal failure (using KDIGO ≥ 2) at day 7	xx.x (xx.x)	xx.x (xx.x)	x.xx (x.xx - x.xx)	x.xx	xx.x (xx.x)	xx.x (xx.x)	x.xx (x.xx - x.xx)	x.xx	x.xx
KDIGO ≥ 2 or death at day 7	xx.x (xx.x)	xx.x (xx.x)	x.xx (x.xx - x.xx)	x.xx	xx.x (xx.x)	xx.x (xx.x)	x.xx (x.xx - x.xx)	x.xx	x.xx
SOFA > 2 at day 3									
Cardiovascular	xx.x (xx.x)	xx.x (xx.x)	x.xx (x.xx - x.xx)	x.xx	xx.x (xx.x)	xx.x (xx.x)	x.xx (x.xx - x.xx)	x.xx	x.xx
Neurologic	xx.x (xx.x)	xx.x (xx.x)	x.xx (x.xx - x.xx)	x.xx	xx.x (xx.x)	xx.x (xx.x)	x.xx (x.xx - x.xx)	x.xx	x.xx
Coagulation	xx.x (xx.x)	xx.x (xx.x)	x.xx (x.xx - x.xx)	x.xx	xx.x (xx.x)	xx.x (xx.x)	x.xx (x.xx - x.xx)	x.xx	x.xx
Respiratory	xx.x (xx.x)	xx.x (xx.x)	x.xx (x.xx - x.xx)	x.xx	xx.x (xx.x)	xx.x (xx.x)	x.xx (x.xx - x.xx)	x.xx	x.xx
Total SOFA score at day 3	xx [xx - xx]	xx [xx - xx]	x.xx (x.xx - x.xx)	x.xx	xx [xx - xx]	xx [xx - xx]	x.xx (x.xx - x.xx)	x.xx	x.xx
SOFA > 2 at day 7									
Cardiovascular	xx.x (xx.x)	xx.x (xx.x)	x.xx (x.xx - x.xx)	x.xx	xx.x (xx.x)	xx.x (xx.x)	x.xx (x.xx - x.xx)	x.xx	x.xx
Neurologic	xx.x (xx.x)	xx.x (xx.x)	x.xx (x.xx - x.xx)	x.xx	xx.x (xx.x)	xx.x (xx.x)	x.xx (x.xx - x.xx)	x.xx	x.xx
Coagulation	xx.x (xx.x)	xx.x (xx.x)	x.xx (x.xx - x.xx)	x.xx	xx.x (xx.x)	xx.x (xx.x)	x.xx (x.xx - x.xx)	x.xx	x.xx
Respiratory	xx.x (xx.x)	xx.x (xx.x)	x.xx (x.xx - x.xx)	x.xx	xx.x (xx.x)	xx.x (xx.x)	x.xx (x.xx - x.xx)	x.xx	x.xx
Total SOFA score at day 7	xx [xx - xx]	xx [xx - xx]	x.xx (x.xx - x.xx)	x.xx	xx [xx - xx]	xx [xx - xx]	x.xx (x.xx - x.xx)	x.xx	x.xx
Mechanical ventilation-free days within 28 days	xx [xx - xx]	xx [xx - xx]	x.xx (x.xx - x.xx)	x.xx	xx [xx - xx]	xx [xx - xx]	x.xx (x.xx - x.xx)	x.xx	x.xx
Tertiary outcomes	xx.x (xx.x)	xx.x (xx.x)	x.xx (x.xx - x.xx)	x.xx	xx.x (xx.x)	xx.x (xx.x)	x.xx (x.xx - x.xx)	x.xx	x.xx
Death in ICU									
Death in hospital	xx.x (xx.x)	xx.x (xx.x)	x.xx (x.xx - x.xx)	x.xx	xx.x (xx.x)	xx.x (xx.x)	x.xx (x.xx - x.xx)	x.xx	x.xx
Days in ICU (days)	xx.x (xx.x)	xx.x (xx.x)	x.xx (x.xx - x.xx)	x.xx	xx.x (xx.x)	xx.x (xx.x)	x.xx (x.xx - x.xx)	x.xx	x.xx
Days in hospital (days)	xx [xx - xx]	xx [xx - xx]	x.xx (x.xx - x.xx)	x.xx	xx [xx - xx]	xx [xx - xx]	x.xx (x.xx - x.xx)	x.xx	x.xx
Dias no hospital (dias)	xx [xx - xx]	xx [xx - xx]	x.xx (x.xx - x.xx)	x.xx	xx [xx - xx]	xx [xx - xx]	x.xx (x.xx - x.xx)	x.xx	x.xx

95%CI - 95% confidence interval; KDIGO - Kidney Disease Improving Global Outcomes; SOFA - Sequential Organ Failure Assessment; ICU - intensive care unit.

* p value for marginal comparison between Plasma-Lyte against 0.9% sodium chloride;

† p value for marginal comparison between slow infusion against rapid infusion;

‡ p value for interaction between saline and infusion speed. Results expressed as n (%) or median [interquartile range].

### Primary outcome analysis

Mortality until 90 days will be tested using mixed-effects Cox proportional hazard models, considering sites as the random variable (frailty models)^(3)^ adjusted for age, baseline SOFA^(4)^ score and the type of admission (planned admission, unplanned admission with baseline sepsis and unplanned admission without baseline sepsis). Sepsis will be defined as infection plus organ failure as per the Sepsis 3 criteria.^(2)^ Proportionality of the hazard ratio will be assessed using the Grambsch and Thernau method,^(5)^ and interactions between the intervention arms (infusion solution and speed) will be tested on this model. If the interaction parameter is significant, we intend to report only one manuscript describing “inside the table” effects, that is, exploring all the four possible combinations and their effects on mortality. If the interaction parameter is not significant at the 5% significance level, marginal effects for each group will be highlighted. In this case, “inside the table” effects shall be reported as supplementary material following CONSORT recommendations for 2 × 2 factorial designs.^(6,7)^ Kaplan-Meier curves will be presented comparing the four arms and separated considering only Plasma-Lyte® *versus* Saline, and Slow *versus* Rapid infusion. Patients with missing follow-up at 90-days will be added to the main primary outcome analysis and censored at their last known follow-up time, patients without discharge information will be imputed by chained equations method using site, age, baseline SOFA score and type of admission.

### Secondary outcomes

The following secondary endpoint analysis were defined:

- Renal replacement therapy up to 90 days.
- Acute renal failure incidence defined as Kidney Disease Improving Global Outcomes (KDIGO)^(8)^ stage 2 or 3 evaluated at days 3 and 7. We plan to use both serum creatinine and diuresis for the KDIGO classification. Diuresis was collected on a daily basis during BaSICS, and therefore we will use the average diuresis over 24 hours as the urinary output criteria in KDIGO; creatinine criteria will be used if diuresis information is not registered. In case of a disagreement between creatinine and diuresis criteria for KDIGO, we will consider the worst criteria.
- SOFA score assessed both as the total value and as individual components and each of their components separately (Cardiovascular, Neurologic, Coagulation, Hepatic and Respiratory) will be evaluated on day 3 and 7.
- Mechanical ventilation-free days within 28 days.

Renal replacement therapy up to 90 days will be estimated using a mixed Poisson model adjusted for age, baseline SOFA and admission type and will be reported as the incidence per 1,000 patient-days. Alternatively, we will also report renal replacement therapy at 90 days in a competing risk model considering death as a competitor for the need for RRT. Incidence of AKI at days 3 and 7 will be tested with mixed generalized linear models with a binomial distribution and the logit link function (also known as a mixed logistic regression model^(9)^) considering site as a random effect; the results will be presented with odds ratios and 95% confidence intervals. Additional analysis combining KDIGO and death will be performed as a supportive analysis to address competitive risks issues.

SOFA score will be tested with a mixed generalized linear model using the distribution that best fits the data (Poisson, gamma, inverse gaussian, or multinomial, among others), and the results will be presented as the mean and/or median differences or ratios with respective 95% confidence intervals using the delta method. Organ dysfunctions at day 3 and 7 (specific SOFA item - cardiovascular, neurologic, coagulation and respiratory as a dichotomous variable defined as higher than 2) will be tested with mixed logistic regression models.

Total SOFA score will be missing if patients died before the measurement time point , and multiple imputation by chain equations using the *mice* R^(10)^ package shall be performed to address the competitive risk bias. Acquired SOFA trend and baseline characteristics will be considered to predict any missed SOFA scores at 3 and 7 days, with the study arms as covariates. Sensitivity analysis without the arms as covariates shall also be performed.

Mechanical ventilation-free days within 28 days will be tested considering the proportion of ventilator-free days in that time frame using zero/one inflated beta or beta-binomial regression assuming zero free-days for those patients who died within that period independently of the amount of time the patient actually used the ventilator. We will consider as ventilation day any day where the patient received any duration of mechanical ventilation. Effect measures shall be presented as the absolute mean difference.

### Tertiary outcomes

We defined tertiary endpoints that should be considered exploratory:

1. Intensive care unit (ICU) and hospital mortality will be tested with mixed logistic regression models considering site as the random effect and adjusted for the same variables used in the primary analysis.
2. Length of stay in the ICU and in the hospital will be compared with mixed generalized linear models with a Poisson distribution and a logarithm link, also considering site as the random intercept effect.
3. Quality of life six months after ICU discharge will be analyzed in a 10% sample using EQ-5D-3L questionnaires.^(11)^ This will be reported separately in a different manuscript.

### Subgroup analyses

Subgroup analyses will be performed with mixed effects Cox proportional hazard models for the primary outcome. We intend to report interaction p-values and hazard ratios of Plasma-Lyte against Saline and Slow against Rapid infusion in each subgroup:

1. Patients with and without sepsis, defined using Sepsis 3 criteria.^(2)^
2. Patients with baseline KDIGO 1 and those ≥ 2.
3. Surgical and nonsurgical patients.
4. Patients with or without traumatic brain injury.
5. Patients with Acute Physiology and Chronic Health Evaluation II (APACHE II)^(12)^ ≥ 25 and < 25 points.
6. Patients who received > 1.000mL *versus* ≤ 1.000mL in the 24 hours before randomization.

### Sensitivity analyses

A per protocol analysis for the primary outcome will also be carried out as a sensitivity analysis, accounting for adherence to the allocated solution and infusion speed. As the per protocol population, we will consider patients meeting all inclusion and exclusion criteria that were infused in at least one allocated group (A to F) a solution bolus at the allocated speed on the first day after randomization. We plan a sensitivity analysis for KDIGO criteria considering only creatinine levels for categorization.

### Protocol adherence

Protocol compliance will be described as presented in tables 3 and 4, reporting the volume of fluids infused on days 1, 2, 3 and 7 comparing Plasma-Lyte 148 or 0.9% saline and the proportion of expansion fluids in the randomized infusion speed (slow or rapid). For the purpose of reporting, any fluid challenge at the incorrect speed will be considered a protocol deviation. The use of normal saline, Lactated Ringer, or other crystalloids above 100mL^(1)^ for diluents or a bolus will also be reported as deviations in the tables.

**Table 3:** Adherence to protocol (for the Plasma-Lyte *versus* 0.9% sodium chloride)3

	Plasma-Lyte		0.9% Sodium chloride		p value
	Patients	Volume received - mL	Patients	Volume received - mL	
Trial fluid					
Day 1	xx (xx.x)	xxxx [xxxx - xxxx]	xx (xx.x)	xxxx [xxxx - xxxx]	x.xx
Day 2	xx (xx.x)	xxxx [xxxx - xxxx]	xx (xx.x)	xxxx [xxxx - xxxx]	x.xx
Day 3	xx (xx.x)	xxxx [xxxx - xxxx]	xx (xx.x)	xxxx [xxxx - xxxx]	x.xx
Day 7	xx (xx.x)	xxxx [xxxx - xxxx]	xx (xx.x)	xxxx [xxxx - xxxx]	x.xx
Open label 0.9% sodium chloride					
Day 1	xx (xx.x)	xxxx [xxxx - xxxx]	xx (xx.x)	xxxx [xxxx - xxxx]	x.xx
Day 2	xx (xx.x)	xxxx [xxxx - xxxx]	xx (xx.x)	xxxx [xxxx - xxxx]	x.xx
Day 3	xx (xx.x)	xxxx [xxxx - xxxx]	xx (xx.x)	xxxx [xxxx - xxxx]	x.xx
Day 7	xx (xx.x)	xxxx [xxxx - xxxx]	xx (xx.x)	xxxx [xxxx - xxxx]	x.xx
Open Label Plasma-Lyte					
Day 1	xx (xx.x)	xxxx [xxxx - xxxx]	xx (xx.x)	xxxx [xxxx - xxxx]	x.xx
Day 2	xx (xx.x)	xxxx [xxxx - xxxx]	xx (xx.x)	xxxx [xxxx - xxxx]	x.xx
Day 3	xx (xx.x)	xxxx [xxxx - xxxx]	xx (xx.x)	xxxx [xxxx - xxxx]	x.xx
Day 7	xx (xx.x)	xxxx [xxxx - xxxx]	xx (xx.x)	xxxx [xxxx - xxxx]	x.xx
Other nontrial crystalloids					
Day 1	xx (xx.x)	xxxx [xxxx - xxxx]	xx (xx.x)	xxxx [xxxx - xxxx]	x.xx
Day 2	xx (xx.x)	xxxx [xxxx - xxxx]	xx (xx.x)	xxxx [xxxx - xxxx]	x.xx
Day 3	xx (xx.x)	xxxx [xxxx - xxxx]	xx (xx.x)	xxxx [xxxx - xxxx]	x.xx
Day 7	xx (xx.x)	xxxx [xxxx - xxxx]	xx (xx.x)	xxxx [xxxx - xxxx]	x.xx
Other nontrial colloids					
Day 1	xx (xx.x)	xxxx [xxxx - xxxx]	xx (xx.x)	xxxx [xxxx - xxxx]	x.xx
Day 2	xx (xx.x)	xxxx [xxxx - xxxx]	xx (xx.x)	xxxx [xxxx - xxxx]	x.xx
Day 3	xx (xx.x)	xxxx [xxxx - xxxx]	xx (xx.x)	xxxx [xxxx - xxxx]	x.xx
Day 7	xx (xx.x)	xxxx [xxxx - xxxx]	xx (xx.x)	xxxx [xxxx - xxxx]	x.xx
Packed red blood cells					
Day 1	xx (xx.x)	xxxx [xxxx - xxxx]	xx (xx.x)	xxxx [xxxx - xxxx]	x.xx
Day 2	xx (xx.x)	xxxx [xxxx - xxxx]	xx (xx.x)	xxxx [xxxx - xxxx]	x.xx
Day 3	xx (xx.x)	xxxx [xxxx - xxxx]	xx (xx.x)	xxxx [xxxx - xxxx]	x.xx
Day 7	xx (xx.x)	xxxx [xxxx - xxxx]	xx (xx.x)	xxxx [xxxx - xxxx]	x.xx

Results expressed as n (%) or median [interquartile range].

**Table 4:** Adherence to the protocol (for the slow versus fast infusion speed comparison)

Speed adherence	Slow infusion speed group	Fast infusion speed group	p value
Day 1			
Patient with at least one bolus infused for expansion	xx/xx (xx.x)	xx/xx (xx.x)	x.xx
All fluid challenges at the allocated speed	xx/xx (xx.x)	xx/xx (xx.x)	x.xx
Maintenance fluid	xx/xx (xx.x)	xx/xx (xx.x)	x.xx
Other infusion	xx/xx (xx.x)	xx/xx (xx.x)	x.xx
Day 2			
Patient with at least one bolus infused for expansion	xx/xx (xx.x)	xx/xx (xx.x)	x.xx
All fluid challenges at the allocated speed	xx/xx (xx.x)	xx/xx (xx.x)	x.xx
Maintenance fluid	xx/xx (xx.x)	xx/xx (xx.x)	x.xx
Other infusion	xx/xx (xx.x)	xx/xx (xx.x)	x.xx
Day 3			
Patient with at least one bolus infused for expansion	xx/xx (xx.x)	xx/xx (xx.x)	x.xx
All fluid challenges at the allocated speed	xx/xx (xx.x)	xx/xx (xx.x)	x.xx
Maintenance fluid	xx/xx (xx.x)	xx/xx (xx.x)	x.xx
Other infusion	xx/xx (xx.x)	xx/xx (xx.x)	x.xx
Day 7			
Patient with at least one bolus infused for expansion	xx/xx (xx.x)	xx/xx (xx.x)	x.xx
All fluid challenges at the allocated speed	xx/xx (xx.x)	xx/xx (xx.x)	x.xx
Maintenance fluid	xx/xx (xx.x)	xx/xx (xx.x)	x.xx
Other infusion	xx/xx (xx.x)	xx/xx (xx.x)	x.xx

Results expressed as n (%).

### Adverse events report

BaSICS will collect only Suspected Unexpected Serious Adverse Reaction (SUSAR) data from the sites.^(1)^ This information will be reported as the number of events per group for both interventions and for the possible four combinations in the study.

### Exploratory analyses

We hope the BaSICS trial will provide relevant information on fluid management in critically ill patients. However, additional analyses that explore potential mechanisms and confounders are planned to aid in study interpretation. These analyses are expected to be reported separately.

- Mean adjusted chloride load (MACL): MACL, defined as total infused chloride over the total volume of infused fluids, is expected to be related to the randomization arm since Plasma-Lyte 148 has 98mEq/L and normal saline has 154mEq/L. It has been suggested that MACL can mediate the effect of saline on outcomes;^(13)^ that is, by using saline, the MACL will be higher (and will approach 154mEq/L if only saline is used), and a higher MACL can cause harm. Based on the daily infusion of fluids used per patient, including all open-label infusions, we will estimate the daily MACL and the cumulative MACL effects on mortality in a time to event analysis for the ICU and for in hospital mortality. We intend to adjust a Cox proportional hazard frailty model and test the incremental effect of the daily and accumulated MACL and total fluid infusion volume as a time-dependent variable until day 3. This analysis will, therefore, consider both the total volume of infused fluids and the MACL and will explore both potential factors on short-term outcomes. Splines will be considered when modeling.
- Chloride subgroup analysis: It is also conceivable that serum chloride may be a driver of organ failure and that part (or all) of the effects of the fluid type intervention is mediated by serum chloride; that is, harms of saline may be more prominent in patients with higher baseline serum chloride. We plan to tackle this issue initially with a sensitivity analysis among patients with low and high (> 110mEq/L) baseline serum chloride for the primary endpoint and the renal secondary endpoints. It should be highlighted that the measurement of serum chloride was not obligatory in BaSICS and therefore not all patients will be available for this analysis.
- Bayesian network for SOFA (total, and all components except hepatic): Death is a competing event for SOFA measurements over time. One approach^(14)^ is to draw Bayesian networks for each intervention arm and to observe transitions to possible states at D2, D3 and D7 according to the randomization group (for both arms) while accounting for death as an absorbent state. This approach can answer, for example, what is the probability that a patient who has a baseline hemodynamic SOFA of 3 and received a slow infusion is alive without vasopressors (alive and SOFA < 3) at day 3? We can obtain relative risks and 95% confidence intervals through bootstrapping the data set. Total SOFA will be categorized for this analysis in quartiles. SOFA components are planned to be used as 5 level categorical variables.

Hemodynamic substudy: A subgroup of ICUs collected blood pressure, central venous pressure, heart rate and several other parameters to address tissue perfusion immediately after every bolus infusion and each half hour until one hour after total infusion of the 500mL bolus. This primary study endpoint was defined as the mean blood pressure within one hour after fluid expansion, focusing on infusion speeds (slow *versus* fast). We intend to use generalized mixed linear models for all continuous variables to address individual repeated measures over time for the intercept and/or slope by infusion, nested with patients without covariates. Mean blood pressure shall be well fitted as a normal distribution.

## CONCLUSION

This manuscript outlines the statistical analytical plan for the BaSICS randomized controlled trial, including all primary, secondary and tertiary objectives. Details of subgroup analyses and potential exploratory analyses are also provided.

## Apêndice 1 DATA Monitoring Committee (DMC) Charter for the BaSICS Trial

**DATA Monitoring Committee (DMC) Charter for the BaSICS Trial**

**November 2017**

**Introduction**

This Charter is for the Data Monitoring Committee (DMC) for the BaSICS (Balanced solution versus Saline in Intensive Care Study - A 2x2 factorial randomized study to evaluate the effect of a balanced crystalloid solution compared with 0.9% saline, and of rapid vs. slow infusion on clinical outcomes of critically ill patients).

The Charter will define the primary responsibilities of the DMC, its membership, and the purpose and timing of its meetings. The charter will also provide the statistical monitoring guidelines to be implemented by the DMC, and an outline of the content of the meetings (both open and closed).

**Responsibilities of the DMC**

1. To help ensure the safety of patients in the trial by protecting them from avoidable harm.
2. To provide the Steering Committee with advice about the conduct of the trial and the integrity of the data, so as to protect the validity and scientific credibility of the trial. In this regard, the DMC may provide suggestions regarding selection, recruitment and retention of participants; study interventions; adherence to protocol-specified regimens; and the procedures for data management and quality control. However, the DMC will have only a limited role on this issue because their detailed review of trial progress will occur only infrequently.
3. To evaluate interim analyses and judge efficacy, harm, and net clinical effect.

**DMC composition**

1. The DMC Chair, Dr. Gordon Guyatt, from Mc Master University, has been invited by the Coordinating Centre.
2. DMC members have been selected by the DMC chair in collaboration with the steering committee for their trial experience plus expertise with intensive care medicine and/or statistics. DMC members are:
  - Niall Ferguson - Intensivist - University of Toronto, Toronto, Canada;
  - Stephen Walter - Statistician - McMaster University, Hamilton, Canada.
3. BaSICS investigators, members of BaSICS Steering Committee and members of the Coordinating Centre have been excluded from the DMC.

**Conflict of interest**

1. DMC members will disclose to the DMC Chair any present conflicts that they consider relevant, and any new conflicts that arise as the study proceeds. The DMC chair will disclose his conflicts, and any conflicts that arise, to the Chair of the Steering Committee, who will judge whether conflicts are of concern.
2. The Steering Committee Chair and DMC Chair have reviewed conflicts and determined that current conflicts will not compromise the DMC members from executing their role disinterestedly.

**Meetings**

Frequency of meetings

1. An initial meeting between the DMC and the BaSICS steering committee early in the trial is planned. Afterwards, DMC will meet to review interim analyses (see “Interim analyses” below).
2. The DMC Chair may request a full meeting of the committee at any time. Conversely, the steering committee may also propose a meeting with the DMC if necessary.

Structure of meetings

1. Initial meeting, with the purpose to finalize the DMC charter, will be open to the steering committee
2. Meetings to review interim analyses will have the following structure:
  - First, an open session with the principal investigator (PI), members of the steering committee, and members of coordinating centre (all of whom remain blinded to treatment specific data) to review accrual, data timeliness and quality, completeness of follow-up, problems with specific centres, and any proposals for changes in the study protocol or study duration. In addition, the PI will be responsible for reporting any new external evidence (especially results from other relevant ongoing trials) that bear on the conduct of the trial. No unblinded information will be revealed during this session.
  - Second, a closed session (PI, steering committee, and coordinating centre members leave) between the DMC and the unblinded independent statistician(s) to review unblinded data on efficacy and safety, and the status of statistical monitoring boundaries (Appendix).
  - Third, an optional executive session may be held with only DMC members present.
  - Lastly, an open session between the DMC and the blinded PI and steering committee will be held to deliver and discuss the DMC comments and recommendations and to decide on the timing of the next meeting. This session may be held by telephone or tele/videoconference.

Minutes

1. The Chair, or someone delegated by the Chair, will take minutes at closed sessions. The PI, or someone delegated by the PI, will take minutes at open meetings. The DMC Statistician will be responsible for archiving the closed session minutes. These will be considered confidential and should be available only for DMC members until the end of the trial.
2. After each meeting the DMC Chair will provide the PI with a letter stating the general outcome of this meeting and suggested changes to the trial conduct. For example, this letter may simply contain the statement that the trial should continue as planned.

**Decisions about stopping the trial**

Based on interim analyses, and, possibly, on external evidence, the Data Monitoring Committee shall decide whether there is evidence beyond a reasonable doubt that the experimental treatment of any of the two comparisons (Plasma-Lyte vs saline, or slow versus rapid fluid administration) is deleterious for all patients or for any subgroup. The DMC may also decide that the accumulating data provides overwhelmingly convincing evidence that the experimental are superior to control treatments (Plasma-Lyte better than saline, or slow better than rapid) and recommend stopping the study for efficacy.

In the event that the DMC recommends one of the trial comparisons (Plasma-Lyte vs Saline, or slow versus rapid) be stopped, they will immediately notify the PI. The DMC will explain the basis of their recommendation to the steering committee and discuss the results together.

If the steering committee, and DMC agree as to the course of action, that is, to stop one of the trial comparisons early, plans will be put into operation for the orderly conclusion of the trial, notification of study patients, and dissemination of the results.

In the unlikely event that the DMC and steering committee members disagree about the proper course of action, the steering committee and DMC will make every attempt to reach a consensus through discussions. If, despite best efforts, significant differences of opinion persist, then additional input from individuals (selected by mutual agreement) will be sought. Every attempt will be made to reach a consensus through this process.

**Interim Analyses**

Role of the coordinating centre and independent statistician

Every effort will be made by the Coordinating Centre to provide the data for interim analyses to the DMC without delay, in order to ensure the safety of patients. A statistician independent from the study investigators will be hired by the coordinating centre to conduct the interim analyses and present them to the Data Monitoring Committee. Results of interim analyses must not be presented to the steering committee, members of study office or any investigators.

**Frequency of interim analysis**

BaSICS is a 2x2 factorial trial assessing two questions:

1. Whether, when compared with 0.9% saline, a balanced crystalloid solution (Plasma- Lyte®) used for plasma expansion may reduce 90-day mortality in critically ill patients.
2. Whether a slow (333 mL/h) compared to a rapid administration (999 mL/h) of crystalloid solution may reduce 90-day mortality in critically ill patients.

While reasonable evidence exists from previous studies suggesting that the effect of balanced versus unbalanced solutions may be small (if any), there is much greater uncertainty regarding the effect size of use of different infusion speeds on clinical outcomes of critically ill patients. For this reason, a safety interim analysis for this comparison early in the course of the study is warranted. Therefore, the following scheme of interim analysis was defined:

- 1000 patients: safety assessment of the fluid speed comparison. The DMC may request additional interim analyses for fluid speed comparisons (for example, for every
- additional 1000 patients).
- 25% of sample size (2750 patients): assessment for both comparisons
- 50% of sample size (5500 patients): assessment for both comparisons
- 75% of sample size (8250 patients): assessment for both comparisons

Stopping boundaries

The DMC will utilize statistical monitoring boundaries as proposed in this charter. These boundaries will be considered guidelines, not rules. Any DMC recommendation should be based on the pattern of all outcomes (efficacy and safety) within the trial and the totality of evidence in existence.

Stopping for safety

If any of the interim analyses shows that any of the experimental interventions (Plasma-Lyte or slow infusion rate) compared to their respective control (0.9% saline or fast infusion rate) is associated with an excess in the 90-day death rate with a two-sided P-value <0.01, this will trigger DMC discussions about stopping the specific comparison for harm. Considering that estimates of effect obtained in interim analyses tend to overestimate true effect, the DMC may request a confirmatory interim analysis after further 500 patients are enrolled.

We opted for fixing the boundaries to guide stopping for safety at a less stringent P-value to protect patients’ safety.

Stopping for efficac*y*

The DMC will adopt much stricter criteria to stop for efficacy than for safety:

1. Stopping for efficacy will be considered only in the interim analyses of 50% and 75% of the total sample size. With larger sample size/number of events the random error should be smaller. Thus, even though size of effect may still be overestimated, the size of bias will tend to be lower.
2. The P-value to consider stopping for efficacy should be <0.001. The DMC may request a confirmatory interim analysis after further 500 or 1000 patients are enrolled to allow for regression of the effect estimates to the mean.

The rationale for stricter criteria to stop for efficacy is summarized in the following arguments:

early discontinuation of randomized trials due to efficacy tends to produce biased estimates of effect (overestimation of the true effect), which may lead to erroneous medical guidelines and decisions; 2) according to the ethical principle of non-maleficence, a new treatment should not be used until there is clear, objective evidence that it is beneficial; 3) clinical practice usually does not change unless there is convincing evidence of the advantages of the new treatment, which would be undermined if the study is discontinued early due to benefits; the decision of early discontinuation of the experimental treatment due to benefits may not be advantageous for future patients.

**Serious Adverse Events**

Serious adverse events which are study related according to the site investigators should be urgently reported (within 24 hours from the onset of the event) to the coordinating centre. Those events will be immediately forwarded to the DMC members. A serious adverse event directly related to the study is defined as any event meeting the two following criteria:

1. Any fatal or life-threatening event (immediate risk of death), or any event that causes sequelae or permanent disability, or that extends hospitalization; AND
2. The primary physician believes that the event is related to the patient’s inclusion in the BaSICS study. Serious adverse events will be considered as “related to the study” if the primary physician believes that the event was probably caused by the fluid and/or rate of infusion used in the study and follows a plausible time sequence after the administration of the fluid.

**Publication Policy**

1. The PI will provide the DMC with a copy of the intended main trial results publication 14 days prior to the intended submission, in order to allow the DMC to review the intended publication and provide input.
2. The DMC will recommend any changes to the publication it reasonably believes are necessary for scientific purposes. The PI and Coordinating Centre agree to thoroughly consider the implementation of all such recommended changes. Notwithstanding the above, the final decision regarding the content of any publication shall be that of the Coordinating Centre.
